# Basic and clinical immunology – 3020. Fish oil supplementation in early infancy modulates developing infant immune responses but not clinical allergy

**DOI:** 10.1186/1939-4551-6-S1-P196

**Published:** 2013-04-23

**Authors:** Nina D'vaz, Manori Amarasekera, Janet Dunstan, Suzanne Meldrum, Tracey Lee-Pullen, Jessica Metcalfe, Barbara Holt, Michael Serralha, Meri Tulic, Trevor Mori, Susan Prescott

**Affiliations:** 1 School of Paediatrics and Child Health, University of Western Australia, Australia; 2 Centre for Microscopy, Characterisation and Analysis, University of Western Australia, Australia; 3 The Telethon Institute for Child Health Research, Centre for Child Health Research, University of Western Australia, Australia; 4 School of Medicine and Pharmacology, University of Western Australia, Australia

## Background

Maternal fish oil supplementation during pregnancy has been associated with altered infant immune responses and a reduced risk of clinical allergy. The objective of this study was to examine the effect of early postnatal fish oil supplementation on infant cellular immune function at six months of age and infant allergic diseases.

## Methods

In a double-blind randomized controlled trial (ACTRN12606000281594), 420 infants of high atopic risk received fish oil (containing 280mg docosahexaenoic acid (DHA) and 110mg eicosapentanoic acid (EPA)) or control oil daily from birth to six months. Fatty acid levels, induced cytokine responses, were assessed at 6 months of age in 150 infants. Eczema, food allergy, asthma and sensitization were assessed in 323 infants who completed clinical follow up at 12 months of age.

## Results

DHA and EPA levels were significantly higher in the fish oil group and erythrocyte arachidonic acid (AA) levels were lower (all p<0.05). Infants in the fish oil group had significantly lower IL-13 responses (p=0.036) to house dust mite and higher IFNg (p=0.035) and TNF (p=0.017) responses to phytohaemaglutinin. Infants with relatively high DHA levels had lower Th2 responses to allergens including lower IL-13 (p=0.020) and IL-5 (p=0.045) to b-lactoglobulin. Although n-3 PUFA levels at 6 months were associated with lower risk of eczema (p=0.033) and recurrent wheeze (p=0.027), the association with eczema was not significant after multiple comparisons. Between group comparisons revealed no differences in the occurrence of allergic outcomes.

## Conclusions

Postnatal fish oil supplementation increased infant n-3 polyunsaturated fatty acid (PUFA) levels and associated with lowered allergen-specific Th2 responses and elevated polyclonal Th1 responses. However, postnatal fish oil supplementation did not prevent childhood allergic disease.

